# Cerebrospinal Fluid in Pediatric Neuro-Oncology: Molecular Diagnosis, Disease Monitoring, and Clinical Translation

**DOI:** 10.3390/ijms27115010

**Published:** 2026-06-01

**Authors:** Aidos Bolatov, Askhat Zhakupov, Malika Sapargaliyeva, Aizhan Abdikadirova, Xingzhi Xu, Mirgul Bayanova

**Affiliations:** 1 Guangdong Key Laboratory for Genome Stability & Disease Prevention and Carson International Cancer Center, Marshall Laboratory of Biomedical Engineering, Shenzhen University Medical School, Shenzhen University, Shenzhen 518060, China; xingzhi.xu@szu.edu.cn; 2 Clinical-Academical Department of Laboratory Medicine, Pathology, and Genetics, “University Medical Center” Corporate Fund, 010000 Astana, Kazakhstan; md.zhakupovaskhat@gmail.com (A.Z.); malika.s@umc.org.kz (M.S.); a.abdikadirova@umc.org.kz (A.A.)

**Keywords:** cerebrospinal fluid, liquid biopsy, pediatric central nervous system tumors, molecular diagnostics, cell-free DNA, circulating tumor DNA, medulloblastoma, diffuse midline glioma, measurable residual disease, precision neuro-oncology

## Abstract

Pediatric brain and other central nervous system (CNS) tumors remain a leading cause of cancer-related death in children, while contemporary management increasingly depends on molecular classification, risk stratification, and longitudinal disease assessment. Yet tissue-based profiling has major limitations in pediatric neuro-oncology, particularly for deep-seated, eloquent, or surgically hazardous tumors and when repeat sampling is impractical. For primary CNS tumors, cerebrospinal fluid is generally more informative than plasma because it is anatomically closer to the tumor and more enriched for tumor-derived material. This narrative review summarizes current and emerging applications of cerebrospinal fluid in pediatric neuro-oncology, from conventional staging to molecular diagnosis, methylation-based classification, measurable residual disease detection, pharmacodynamic monitoring, and relapse surveillance. We discuss the biological rationale for cerebrospinal fluid analysis, major pre-analytical and technical determinants of assay performance, and the strengths and limitations of key analyte classes, including cytology, circulating tumor cells, cell-free DNA, RNA, extracellular vesicles, proteins, and metabolites. We also summarize how these approaches are being applied across major pediatric central nervous system tumor entities. Cerebrospinal fluid liquid biopsy is unlikely to replace tissue or imaging, but is increasingly positioned to complement both in precision pediatric neuro-oncology.

## 1. Introduction

Pediatric brain and other central nervous system (CNS) tumors remain a major cause of cancer-related morbidity and mortality. Population-based data consistently show that they are the most common solid tumors of childhood and adolescence and the leading cause of cancer death in this age group, underscoring the need for diagnostic strategies that are both biologically informative and clinically feasible [1]. At the same time, pediatric neuro-oncology has undergone a fundamental shift from morphology-centered diagnosis toward integrated molecular classification, in which genomic and epigenomic features increasingly shape tumor definition, risk stratification, and therapeutic decision-making [1,2].

Yet the clinical implementation of molecular neuro-oncology still depends predominantly on tumor tissue obtained at surgery or biopsy. Although tissue remains the diagnostic gold standard, tissue-based assessment has important limitations in children. Neurosurgical sampling may be difficult, hazardous, or intentionally deferred in deep-seated, midline, or eloquent lesions; small specimens may be inadequate for comprehensive downstream profiling; and even when tissue is available, it represents only a single spatial and temporal snapshot of a biologically evolving disease [2,3]. These constraints are especially relevant in pediatric CNS tumors, where repeat sampling is often impractical and where timely molecular information may directly alter diagnosis and treatment selection.

Against this background, cerebrospinal fluid (CSF) has become a particularly informative liquid-biopsy compartment for primary CNS tumors. Because CSF is anatomically contiguous with the brain and spinal cord and lies in close proximity to intracranial disease, it is generally more informative than plasma for tumor-derived material in CNS malignancies [3,4,5]. This relative enrichment reflects both compartmental proximity and the limited transfer of tumor-derived nucleic acids across the blood–brain barrier into the systemic circulation [3,4]. As a result, CSF can provide access not only to cell-free DNA (cfDNA) and circulating tumor DNA (ctDNA), but also to circulating tumor cells (CTCs), RNA species, extracellular vesicles (EVs), proteins, and metabolites, thereby offering a multidimensional molecular window into pediatric brain tumors [3,5].

Historically, CSF was used primarily for staging, most notably for cytological assessment of leptomeningeal dissemination. That role remains clinically important, but it no longer captures the full diagnostic potential of the compartment. Recent studies show that CSF-derived analytes can support mutation detection, copy-number profiling, methylation-based classification, and longitudinal disease monitoring across multiple pediatric CNS tumor types [3,6,7,8]. In embryonal tumors, low-pass whole-genome sequencing (LP-WGS) of CSF-derived cfDNA has demonstrated greater sensitivity than conventional cytology for tumor detection at staging while enabling serial assessment of genomic evolution [6,8]. More recent methylation-based studies have extended CSF analysis beyond mutation detection toward molecular classification itself, including in low-input and real-world pediatric samples, and have further highlighted the feasibility of CSF-derived DNA methylation profiling for CNS tumor classification [7,9,10]. Taken together, these studies show that CSF has uses beyond cytological staging, but as a biologically privileged liquid-biopsy compartment with expanding relevance for diagnosis, classification, and disease monitoring in pediatric neuro-oncology [3,6,7,8,9,10].

In this review, we examine the evolving clinical role of CSF in pediatric brain tumors, from its established use in staging to its emerging applications in molecular diagnosis, tumor classification, longitudinal monitoring, and precision neuro-oncology. We focus specifically on the biological rationale for CSF analysis, the pre-analytical and technical determinants of assay performance, the major CSF analyte classes, tumor-specific applications, and the clinical decision points at which CSF liquid biopsy is most likely to affect patient care. The conceptual contribution of this review is to frame CSF liquid biopsy not as a broadly applicable replacement for tissue, imaging, plasma, or conventional cytology, but as a decision-dependent translational tool. We synthesize current evidence into a clinical utility framework that considers tumor–CSF anatomy, expected shedding biology, analyte class, procedural feasibility, and management relevance. This approach allows CSF-based assays to be evaluated according to when they provide information that cannot be reliably obtained from less invasive or already established methods, and when that information is likely to alter diagnosis, risk stratification, treatment selection, monitoring, relapse detection, or trial eligibility.

### Literature Search Strategy

This article is a narrative review intended to synthesize current evidence and translational concepts rather than to provide a systematic review or meta-analysis. Relevant literature was identified through searches of PubMed/MEDLINE, Web of Science, Scopus, and Google Scholar, supplemented by manual screening of reference lists from selected primary studies, review articles, and clinical reports. The literature search was last updated on 26 May 2026. Eligible sources included peer-reviewed publications available online by the final search date, including articles published online ahead of print. We placed particular emphasis on studies published during the past decade, while also including earlier clinically relevant work on conventional CSF cytology, staging, and established tumor markers.

Search terms included combinations of: “cerebrospinal fluid”, “CSF”, “liquid biopsy”, “circulating tumor cell”, “CTC”, “cell-free DNA”, “cfDNA”, “circulating tumor DNA”, “ctDNA”, “DNA methylation”, “methylation profiling”, “RNA”, “microRNA”, “extracellular vesicles”, “proteomics”, “metabolomics”, “pediatric brain tumor”, “pediatric CNS tumor”, “medulloblastoma”, “diffuse midline glioma”, “DIPG”, “pediatric high-grade glioma”, “ependymoma”, “atypical teratoid/rhabdoid tumor”, “AT/RT”, “pediatric low-grade glioma”, and “intracranial germ cell tumor”. Additional searches used terms related to “measurable residual disease”, “minimal residual disease”, “molecular monitoring”, “leptomeningeal dissemination”, “pre-analytical variables”, “blood contamination”, “CSF sampling”, and “clinical implementation”.

We prioritized original studies, clinically relevant cohort studies, translational reports, technical validation studies, and high-quality reviews addressing CSF-based biomarkers in pediatric CNS tumors or directly applicable CNS tumor liquid-biopsy contexts. Studies were included when they provided information on biological rationale, analyte detectability, assay performance, tumor-specific applications, pre-analytical determinants, or clinical decision points. Adult CNS tumor and leptomeningeal-disease studies were included selectively when pediatric data were limited and the findings were mechanistically or technically relevant to CSF liquid biopsy. Non-peer-reviewed material, purely speculative reports, and studies without clear relevance to CSF-based tumor assessment were generally excluded. Because this was not a systematic review, no formal risk-of-bias assessment or quantitative evidence grading was performed.

## 2. Current Clinical Uses of CSF in Pediatric Neuro-Oncology

CSF is already an established clinical specimen in pediatric neuro-oncology, even before consideration of modern liquid-biopsy technologies. Its most established role is in dissemination assessment, where postoperative lumbar CSF cytology is interpreted alongside neuraxis MRI to evaluate leptomeningeal spread in tumors with recognized seeding potential, particularly medulloblastoma (MB), ependymoma, atypical teratoid/rhabdoid tumor (AT/RT), and selected intracranial germ cell tumors (iGCTs) [11,12,13,14,15,16,17,18,19,20,21,22,23,24]. In MB, positive CSF cytology remains clinically important because it corresponds to M1 disease and can alter risk assignment and adjuvant treatment intensity, including the use and dose of craniospinal irradiation [11,12,18,21,22,25]. In ependymoma and AT/RT, the role of CSF is more selective and entity-dependent, but positive findings may still influence dissemination staging and treatment intensification [13,14,15,16]. Thus, CSF already has an established role in formal disease assessment across several pediatric CNS tumor types (Table 1).

CSF is also used for established disease-specific biochemical markers, showing that its clinical role extends beyond cytology alone. This is most evident in iGCTs, where CSF alpha-fetoprotein (AFP) and beta-human chorionic gonadotropin (β-hCG) are integrated into diagnosis, disease classification, risk stratification, and response assessment during treatment. In this setting, CSF can function as a clinically actionable biochemical specimen that helps distinguish germinoma from non-germinomatous germ cell tumor (NGGCT) and supports response assessment during therapy [24,27,28]. This precedent is important because it shows that molecular CSF applications extend an existing clinical role rather than create a completely new one.

At the same time, the informational range of current CSF practice remains limited relative to the needs of modern pediatric neuro-oncology. Conventional CSF assessment is strongest for dissemination staging and selected biochemical applications, but much weaker for molecular classification, treatment-response assessment, minimal residual disease detection, and resistance tracking [12,14,15,26]. Its performance is also shaped by sampling compartment and perioperative context, with lumbar, ventricular, and shunt-derived specimens not being diagnostically equivalent, and early postoperative samples particularly vulnerable to blood contamination or procedure-related confounding [33,34,35,36]. These limitations do not diminish the clinical importance of current CSF practice; they define why molecular augmentation of CSF has become increasingly important [3,6,8,37].

To understand why CSF is particularly well-suited to this expanded role, it is first necessary to consider the biological features that distinguish it from peripheral blood as a tumor-associated biofluid.

## 3. Biological Rationale for CSF-Based Tumor Assessment

**Figure 1:**
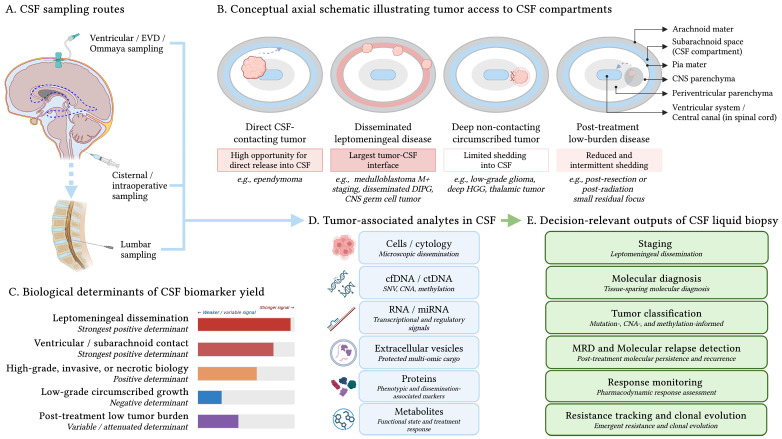
From tumor–CSF interface to clinical information: a conceptual framework for CSF liquid biopsy in pediatric neuro-oncology. The figure summarizes the biological and translational logic that underpins CSF liquid biopsy in pediatric CNS tumors. (**A**) Different CSF sampling routes interrogate distinct biological compartments. Lumbar, ventricular, cisternal, shunt-derived, external ventricular drain (EVD)-derived, Ommaya-derived, and intraoperative specimens are not biologically interchangeable because they differ in tumor proximity, CSF flow dynamics, and background cellular or protein content. (**B**) Tumor state and anatomical relationship to CSF spaces shape analyte recovery. Tumors directly contacting ventricular or subarachnoid surfaces, as well as leptomeningeal/disseminated disease, are expected to yield stronger CSF signal, whereas deep non-contacting circumscribed tumors and post-treatment low-burden states typically produce weaker or more intermittent signal. (**C**) Biological determinants of CSF biomarker yield include the extent of tumor–CSF interface, leptomeningeal dissemination, high-grade/invasive/necrotic biology, low-grade circumscribed growth, and treatment-related reduction in tumor burden. (**D**) These conditions influence which tumor-associated analytes are recoverable from CSF, including cells/cytology, cfDNA/ctDNA, RNA/miRNA, extracellular vesicles, proteins, and metabolites. (**E**) In turn, these analytes can support clinically relevant outputs including staging, tissue-sparing molecular diagnosis, tumor classification, measurable residual disease detection, response monitoring, relapse detection, and resistance/clonal evolution analysis. Overall, the figure illustrates that CSF liquid-biopsy performance depends on tumor anatomy, shedding biology, and clinical context. Created in BioRender. Asanova, A. (2026) https://BioRender.com/jve62dc (accessed on 27 April 2026).

This anatomical relationship provides the biological basis for CSF liquid biopsy in pediatric CNS tumors and explains why CSF is often a preferred liquid-biopsy matrix for primary CNS malignancies [3,38,39]. Rather than restating the general CSF-versus-plasma rationale, this section focuses on the biological factors that determine whether a CSF sample is likely to contain informative tumor-derived signals including tumor–CSF interface, dissemination pattern, tumor grade, necrosis, and treatment context (Table 2, Figure 1A–C).

However, the biological informativeness of CSF is not uniform across tumors. Signal intensity is shaped by the extent of tumor contact with ventricular, subarachnoid, or leptomeningeal spaces, whereas deeply parenchymal or circumscribed lesions are less likely to yield detectable tumor-derived material [5,14,40,50]. Disseminated disease further amplifies this signal by increasing the tumor surface exposed to CSF pathways, particularly in embryonal tumors with neuraxial spread and other leptomeningeal-disseminating entities [11,43]. Tumor biology also shapes yield: high-grade, invasive, and necrotic tumors typically release more cfDNA and protein than indolent low-grade lesions, whereas post-treatment low-burden states may generate only sparse or intermittent signal despite persistent disease [30,37,44,45,46,47,48,49]. Consequently, CSF liquid biopsy should be interpreted in relation to tumor anatomy, dissemination pattern, treatment context, sampling route, assay platform, and clinical question.

These determinants also frame the interpretation of positive and negative CSF findings. A positive result may indicate close tumor proximity to CSF spaces, overt dissemination, high cellular turnover, residual disease, or emerging relapse biology. Conversely, a negative result does not exclude disease and may reflect low shedding, limited CSF contact, low tumor fraction, or treatment-related suppression of analyte release [5,14,46,47,48,49,51]. Thus, CSF provides a biologically meaningful but selective view of tumor burden rather than a universal surrogate across pediatric CNS tumors (Figure 1B,C).

CSF is therefore best viewed as a compartment-specific readout whose clinical value depends not only on analytical performance but also on how, when, and from where the sample is obtained [50,52,53]. This framework, linking tumor–CSF interface, sampling compartment, analyte recovery, and downstream clinical use, is summarized in Figure 1.

## 4. Pre-Analytical and Technical Determinants of CSF Biomarker Performance

The performance of CSF biomarkers in pediatric neuro-oncology depends not only on tumor biology and assay sensitivity, but also on how specimens are obtained, processed, stored, and matched to the intended analytical platform. This is particularly important in children, where CSF samples are often low-volume, low-input, and low-tumor-fraction. Under these conditions, small differences in collection, processing, storage, or assay selection may determine whether a low-level tumor signal is detectable [6,8,9]. Accordingly, pre-analytical variation is often a primary determinant of analytical success and interpretability.

A first major layer of variability arises at specimen acquisition. CSF compartments are not biologically interchangeable: lumbar, ventricular, cisternal, shunt-derived, external ventricular drain-derived, Ommaya-derived, and intraoperative samples differ in tumor proximity, CSF flow dynamics, and background cellular or protein composition (Figure 1A). Earlier pediatric cytology studies already showed that lumbar CSF is more informative than shunt-derived CSF for detecting leptomeningeal disease, while intracranial and lumbar compartments may differ in their representation of disseminated disease [33,35]. Similar principles likely apply to molecular analytes, and more recent standardization-oriented work emphasizes that ventricular, lumbar, and device-derived samples should not be pooled without explicit stratification [50]. Timing is equally important. Postoperative samples obtained too early may contain blood, inflammatory debris, or surgically displaced cells and nucleic acids, whereas later samples may better reflect residual disease biology. In MB, formal cytologic staging is most reliable when lumbar puncture is performed approximately 14 days after surgery and not in the immediate postoperative interval [34,36]. Treatment context also matters: radiotherapy and systemic therapy may transiently alter tumor-cell turnover and analyte release, making isolated on-treatment measurements harder to interpret than serial trends obtained at defined milestones [6,9,54]. In pediatric cohorts, specimen volume is an additional constraint because limited CSF reduces the total recoverable amount of DNA, RNA, EVs, proteins, and intact cells, thereby increasing assay failure and false-negative risk, particularly in low-shedding or post-treatment disease states [6,8,9].

A second layer of variability is introduced during processing and storage. Initial aliquots may be disproportionately affected by traumatic blood contamination, which can distort downstream DNA-, RNA-, protein-, and EV-based readouts [55,56]. Centrifugation strategy also reshapes the measurable analyte: whether a sample is left unspun, pre-cleared, or divided into pellet and supernatant materially alters the relative abundance of cells, leukocyte-derived background DNA, cfDNA, EVs, and soluble proteins. For DNA-based profiling, CSF supernatant is generally more informative than cell-pellet DNA, which is often enriched for non-tumor cellular material [57]. Processing delay further affects analyte integrity by promoting cell lysis, release of background genomic DNA, RNA degradation, and EV instability [55,58,59]. Storage conditions and repeated freeze–thaw cycles introduce additional variability, with especially strong effects on RNA, EVs, proteins, metabolites, and intact cells, but also on low-level cfDNA signals near the limit of detection [55,58,59,60]. Thus, the measured analyte is not simply extracted unchanged from the specimen; it is partly shaped by the workflow applied after collection.

A third layer is assay matching. Even an optimally collected specimen may produce an uninformative result if the analytical platform is not aligned with the tumor’s molecular architecture. Recovery efficiency varies across extraction chemistries, particularly at picogram- or subnanogram-level input, and library preparation strategies differ in their tolerance of fragmented or sparse material [8,9,61]. This is especially relevant in pediatric CNS tumors, where hotspot-driven entities such as DMG are well-suited to targeted variant assays, whereas copy-number–rich embryonal tumors are often better matched to LP-WGS, and methylation-based classifiers require the preservation of fragment and epigenetic integrity at very low input [6,8,10]. The same principle extends across analyte classes: performance depends not only on assay sensitivity, but on whether the specimen, workflow, and platform are suited to the biological question being asked [57,58,59,60].

Taken together, CSF biomarker performance is shaped by a chain of interacting variables spanning acquisition, treatment context, processing, storage, and assay design. The practical implication is straightforward: robust clinical translation will require explicit standardization and reporting of the full pre-analytical workflow. Without this, cross-study comparison remains difficult and negative results may reflect technical as much as biological constraints.

## 5. CSF Analyte Classes in Pediatric Brain Tumors

Against this pre-analytical backdrop, the value of CSF depends on which analyte class is interrogated and what biological or clinical information it can provide. These analytes differ in both the information they provide and their current level of clinical maturity. Conventional cytology and chemistry are already embedded in routine care but offer limited molecular resolution. In contrast, cfDNA/ctDNA has emerged as the most clinically advanced molecular analyte class, with demonstrated utility for mutation detection, copy-number profiling, methylation-based classification, measurable residual disease (MRD) assessment, and longitudinal monitoring in selected pediatric CNS tumors [2,6,8,9,38,62,63]. Other analyte classes include CTCs, RNA/miRNA, EVs, proteins, and metabolites, which extend CSF liquid biopsy beyond genomics but remain less standardized, and in most pediatric settings, less clinically mature [3,5,59]. The major analyte classes currently recoverable from CSF are summarized schematically in Figure 1D.

### 5.1. Conventional CSF Assessment: Cytology and Established Markers

Conventional CSF assessment remains clinically relevant because it is rapid, widely available, and already incorporated into pediatric neuro-oncology workflows (Table 3). Biologically, however, it provides only limited resolution. Cytology can identify overt malignant cells in the CSF compartment, but it is fundamentally a morphologic assay and therefore cannot define tumor molecular subtype, capture genomic or epigenomic features, or sensitively track molecular disease dynamics [17,18,20,21,22,64,65,66,67,68]. Its performance is also constrained by low or intermittent tumor-cell shedding, rapid cellular degeneration, sampling geometry, and observer dependence [26,68].

Routine biochemical parameters such as protein, glucose, and cell count are similarly supportive but nonspecific. Their clearest disease-specific role remains in iGCTs, where CSF AFP and β-hCG provide clinically actionable diagnostic and monitoring information [24,71,72]. In contrast, older markers such as lactate dehydrogenase (LDH), β-glucuronidase, and β2-microglobulin have shown limited and inconsistent value and have largely been superseded by more specific molecular approaches [73,74,75]. Thus, conventional CSF assessment remains an important clinical foundation, but its analytes are low-resolution compared with modern molecular platforms.

### 5.2. Cell-Based and DNA-Based Analytes: CTCs and cfDNA/ctDNA

CTCs and cfDNA/ctDNA represent the two most direct routes by which CSF liquid biopsy can recover tumor-derived material. CTCs preserve intact cells and therefore offer, in principle, the richest information content, including cell counts, immunophenotype, and potential single-cell genomic or transcriptomic characterization [76,77,78]. In adult epithelial leptomeningeal metastasis, platform-based CSF CTC assays have repeatedly outperformed conventional cytology and shown diagnostic and prognostic relevance [77,78,80,81,82]. In pediatric neuro-oncology, however, clinical translation remains limited because the most validated systems are EpCAM-based and therefore biologically mismatched to most pediatric primary CNS tumors, which are non-epithelial neuroectodermal neoplasms [76,77,79]. For pediatric tumors, the concept is biologically plausible and supported indirectly by robust tumor shedding into CSF [24,56,60], but true platform-based CTC capture remains largely investigational and will likely require EpCAM-independent, lineage-appropriate strategies [82,83,84,85,86,87].

In contrast, cfDNA/ctDNA is currently the most mature CSF analyte class in pediatric brain tumors. In this review, cfDNA refers to the total cell-free DNA fraction recovered from CSF, whereas ctDNA refers specifically to the tumor-derived component of cfDNA, inferred or confirmed through tumor-specific mutations, copy-number alterations (CNAs), methylation patterns, or other tumor-associated molecular features. This maturation has been enabled in part by the fact that CSF generally outperforms plasma for molecular detection in primary CNS tumors, including pediatric disease [42,88,115,116]. Its clinical strength lies in its compatibility with low-input workflows and in its ability to support multiple assay types within the same specimen. Targeted variant detection, particularly by droplet digital PCR (ddPCR), is well-suited to tumors defined by recurrent hotspot alterations, most clearly H3K27M diffuse midline glioma (DMG), where serial CSF ctDNA tracking has shown clinical-trial-level relevance for response monitoring and early progression detection [90,91,92]. Targeted sequencing panels broaden this approach by enabling mutation, fusion, and copy-number profiling in clinically actionable settings, including MB, high-grade glioma (HGG), and selected low-grade glioma (LGG) [2,38,62,93,94,95]. LP-WGS is especially useful in tumors dominated by recurrent CNAs, particularly embryonal tumors such as MB. In the SJMB03-associated study, serial CSF cfDNA profiling by LP-WGS provided one of the clearest demonstrations that CSF liquid biopsy can function as an MRD platform in a pediatric solid CNS tumor [63]. More recent real-world and multi-entity pediatric studies have confirmed that integrated workflows combining low-pass genome-wide profiling with targeted sequencing can recover complementary information from a single limited-volume CSF sample [6,8,9,96,97].

### 5.3. RNA, miRNA, Extracellular Vesicles, Proteins, and Metabolites

Beyond DNA, CSF contains analytes that capture additional layers of tumor biology, including transcriptional activity, post-transcriptional regulation, vesicle-mediated signaling, secreted proteins, and metabolic state [59,98]. Among these, RNA and miRNA are attractive because they can reflect viable tumor-cell states and pathway activity, while cfDNA more often reflects apoptotic shedding. However, they are also more technically fragile. Much of the stable extracellular RNA signal is carried within EVs or protein complexes, and pediatric studies increasingly suggest that the extracellular small-RNA landscape differs from adult paradigms, with informative contributions from Y RNAs and other noncanonical small RNAs in addition to miRNAs [5,59,98,99,100,101,102]. This is exemplified in MB, where integrated CSF transcriptomic, metabolomic, and lipidomic profiling identified coordinated tumor-associated molecular signatures [99].

EVs are especially attractive because they package multiple information layers simultaneously, including RNA, DNA, proteins, and lipids [103,104]. Studies in glioma have shown that EV fractions can preserve tumor-specific mutations and amplifications [105,106,107], and pediatric work has begun to identify disease-relevant EV-associated proteins such as transketolase in MB with leptomeningeal dissemination [108]. Proteomic and metabolomic CSF biomarkers are similarly compelling because they may reflect secreted tumor phenotypes and real-time tumor physiology. Pediatric proteomic studies have identified candidate markers in MB and other CNS tumors [109,110], while newer work suggests that patient-specific peptide tracking may become feasible for individualized surveillance [111]. Metabolomics has shown that malignant CNS tumors leave detectable metabolic signatures in CSF [112,113], and in pediatric DMG, the ONC201 experience established an important precedent for CSF metabolomics as a pharmacodynamic biomarker platform [114].

Taken together, these non-DNA analytes broaden the informational scope of CSF beyond static genomic profiling. Their biological promise is substantial, but current pediatric evidence remains less mature and less standardized than for cfDNA/ctDNA. Their relative value depends on the tumor context and intended clinical use case (Figure 1D,E).

Across analyte classes, the major translational gap is not only analytical sensitivity, but reproducibility across platforms, thresholds for clinically meaningful positivity, and evidence that a CSF result changes management beyond imaging, cytology, plasma, or tissue-based testing.

## 6. Tumor-Specific Applications of CSF Liquid Biopsy in Pediatric Brain Tumors

The clinical value of CSF liquid biopsy is not uniform across pediatric CNS tumors, but is greatest in entities that combine close anatomical access to CSF spaces, a propensity for neuraxial dissemination, and tractable molecular alterations. Embryonal tumors, particularly medulloblastoma, and DMG/pHGG represent the clearest current use cases for CSF ctDNA-based clinical translation, whereas selected GCTs, pLGG, ependymoma, and rarer embryonal tumors remain more context-dependent applications [38,63,117] (Table 4). For consistency with the narrative organization of this section, Table 4 is grouped by tumor family rather than strictly ranked by strength of ctDNA evidence. This disease-specific pattern is summarized in Figure 1, where tumor anatomy and shedding biology determine analyte recovery and the most realistic clinical use case (Figure 1C–E). To synthesize the biological, analyte-specific, tumor-specific, and translational considerations discussed above, Table 4 integrates the most relevant CSF analytes, clinical use cases, molecular targets, evidence maturity, and current readiness for clinical implementation across major pediatric CNS tumor contexts.

### 6.1. Embryonal Tumors

Among embryonal tumors, MB has the most mature evidence base because it combines CSF access, dissemination potential, and recurrent molecular features that are recoverable from CSF. The dominant current application is cfDNA-based molecular monitoring, particularly subgroup-informed profiling and MRD assessment, with support from mutation, copy-number, and methylation-based approaches [38,119,120]. The main translational gap is no longer proof-of-concept, but prospective standardization within cooperative-group frameworks [63,118,125].

AT/RT is also biologically well-suited to CSF analysis because neuraxial dissemination is common and the defining *SMARCB1*-associated abnormality is more specific than cytology alone. The strongest current use case is cfDNA-based diagnostic clarification and serial monitoring, with early evidence that chromosome 22/*SMARCB1*-associated changes can be detected in CSF and may re-emerge before overt radiographic progression [63,126,127,128]. However, AT/RT remains in an early translational phase, and prospective validation is still needed before routine clinical use.

Embryonal tumor with multilayered rosettes (ETMRs) is distinctive because its defining C19MC biology points directly to an RNA-based biomarker strategy. The most compelling current analyte is tumor-associated miRNA, particularly miR-517a, while emerging multi-omics work suggests that ETMRs may best be approached through integrated RNA-, metabolite-, and DNA-based CSF profiling [129,130,131,132,133,134]. The principal gap is that most supporting evidence remains early-stage, and prospective CSF validation is only now entering trial infrastructure.

Pineoblastoma and other rare embryonal tumors remain evidence-limited, but their anatomical proximity to CSF pathways and dissemination potential make them biologically plausible CSF diseases. The most realistic current application is exploratory cfDNA-based profiling or surveillance in rare-tumor contexts, including distinction from radiologic mimics such as germinoma [9,63]. For these entities, the main need is systematic prospective biobanking rather than immediate disease-specific implementation [117].

### 6.2. Pediatric Gliomas

In pLGG, CSF liquid biopsy is biologically constrained by low shedding, low mutational burden, and often limited tumor–CSF contact. Its most credible current role is tissue-sparing molecular diagnosis in surgically difficult tumors, particularly when actionable MAPK-pathway drivers such as *KIAA1549::BRAF* or *BRAF* V600E would alter treatment [9,42,95]. The main limitation is sensitivity: pLGG is not a broadly informative CSF-monitoring disease, and its utility remains selective even though targeted diagnostic successes have now been reported [8,94,95,135,136,137].

DMG represents the most advanced glioma application because its midline anatomy, limited re-biopsy feasibility, and recurrent H3K27M biology create a clear need for serial molecular assessment. Here, H3K27M ctDNA is the dominant analyte, and the strongest current use case is pharmacodynamic monitoring, including early progression detection and clarification of pseudo-progression during therapy [90,140]. Recent work in diffuse brainstem tumors further supports the feasibility of CSF-based detection of H3K27M and selected actionable alterations such as *BRAF* V600E, although clinical implementation remains selective and investigational [141]. Recent DMG leptomeningeal-disease data also suggest that CSF *H3F3A* K27M-mutant ctDNA may support molecular diagnosis of leptomeningeal dissemination and may be more sensitive than conventional approaches in selected cases, while still requiring integration with imaging and clinical context [142]. Broader pHGG applications are also emerging through targeted sequencing and combined low-pass whole-genome/panel-based workflows, but DMG remains the clearest example of clinically meaningful CSF molecular monitoring in pediatric glioma [8,62,92].

### 6.3. Ependymoma and Intracranial Germ Cell Tumors

In ependymoma, the biological rationale for CSF analysis is strong because the disease already has an established CSF staging framework and because recurrent molecular risk features, particularly 1q gain in posterior fossa group A (PFA) disease, are theoretically well-suited to cfDNA-based detection. The dominant current use case is molecular risk-state tracking, especially at recurrence, with early studies showing that 1q gain and additional chromosomal abnormalities can be detected directly in CSF [37,143,144,145,146]. The main limitation is maturity: ependymoma remains a strong biological case awaiting prospective validation, and for supratentorial ZFTA fusion-positive disease, RNA-based CSF strategies are still largely investigational [144,146].

In contrast, iGCTs already provide the most established CSF biomarker framework in pediatric neuro-oncology because AFP and β-hCG are integral to diagnosis, risk stratification, and treatment monitoring [24,147]. The emerging extension is multi-analyte CSF profiling, particularly ctDNA for marker-equivocal disease or residual-disease assessment, with metabolomic and miRNA-based approaches representing plausible next steps [54,148,149,150]. Thus, the major translational question in iGCT is not whether CSF matters, but how best to expand a mature conventional biomarker model into a broader molecular framework [149,150].

Overall, CSF liquid biopsy shows a disease-specific gradient of maturity, with strongest utility in tumors where anatomy, shedding biology, and molecular targets support a defined clinical use case.

## 7. Clinical Applications of CSF Liquid Biopsy in Pediatric Brain Tumors: A Decision-Point Framework

### 7.1. When Is CSF Liquid Biopsy Justified?

CSF liquid biopsy is transitioning from a predominantly exploratory method to a clinically relevant adjunct in pediatric neuro-oncology. Its value rests on a consistent biological advantage over plasma: because many primary CNS tumors are anatomically contiguous with the CSF compartment, CSF is typically enriched for tumor-derived nucleic acids and yields higher detection rates than blood across multiple pediatric brain tumor types [42,90,137]. This enrichment makes CSF the preferred liquid-biopsy substrate when the clinical question concerns intracranial disease rather than systemic dissemination.

The central translational question is therefore not whether CSF can contain tumor-derived molecular information, but when that information justifies an invasive procedure. CSF liquid biopsy should not be viewed as a universal replacement for tissue diagnosis, MRI surveillance, plasma-based liquid biopsy, or conventional CSF cytology. Its clinical justification is strongest when it provides actionable information that is unavailable, unreliable, or insufficiently specific through less invasive or already established approaches. This includes situations in which tissue biopsy is unsafe or inadequate, plasma is expected to have low sensitivity, MRI cannot distinguish progression from treatment effect, cytology lacks molecular resolution, or serial CSF profiling may identify MRD, molecular response, relapse, or resistance earlier than conventional assessment. Conversely, when high-quality tissue is available, imaging is definitive, or plasma-based testing can answer the clinical question with adequate sensitivity, CSF sampling should be considered only if the expected molecular gain is likely to affect diagnosis, risk stratification, trial eligibility, treatment selection, or surveillance strategy.

Assay choice should follow the clinical question rather than biomarker availability alone. Targeted assays, including ddPCR and focused hybrid-capture NGS, are best suited to mutation confirmation and tissue-sparing molecular diagnosis, particularly when ctDNA burden is low. Broader approaches such as LP-WGS are more useful for copy-number-based MRD assessment, longitudinal surveillance, and relapse tracking once a tumor-specific genomic signature is known [62,151]. At the same time, most applications should still be interpreted as complementary to tissue and imaging rather than as stand-alone replacements, because prospective validation and entity-specific thresholds remain incomplete for many decision points.

A further distinction is needed between technical detectability, analytical validity, and clinical utility. Detecting tumor-derived DNA, RNA, proteins, metabolites, EVs, or cells in CSF demonstrates biological feasibility, but does not by itself establish clinical actionability. Analytical validity requires reproducible performance, acceptable sensitivity and specificity, input tolerance, and inter-platform comparability under defined pre-analytical conditions. Clinical utility requires evidence that the result changes diagnosis, risk assignment, treatment selection, response assessment, relapse detection, trial eligibility, or surveillance beyond existing methods. Because many pediatric CSF liquid-biopsy studies remain proof-of-concept, retrospective, single-center, or based on small cohorts, detectable CSF signal should not be interpreted as a clinically validated biomarker unless analytical robustness and management-relevant benefit have been shown.

The risk–benefit balance is especially important in children because CSF sampling is not equivalent to venipuncture. Lumbar puncture may require sedation or anesthesia, may be contraindicated in the setting of mass effect or raised intracranial pressure, and may be difficult to repeat for longitudinal monitoring. Repeated CSF sampling also raises ethical considerations related to procedural burden, discomfort, sedation exposure, parental consent, age-appropriate assent, and proportionality between anticipated molecular benefit and procedural risk. Ventricular, shunt-derived, Ommaya-derived, EVD-derived, and intraoperative specimens further depend on neurosurgical access, device presence, and sampling timing. CSF analysis is therefore most defensible when it is opportunistic, clinically anchored, and decision-linked: obtained during a clinically indicated procedure when possible, used to answer a question not reliably addressed by tissue, plasma, MRI, or cytology, and expected to change diagnosis, risk assignment, treatment selection, response assessment, relapse detection, or trial eligibility. Conversely, CSF sampling should not be performed solely for exploratory molecular profiling when less invasive or already available methods can adequately answer the clinical question [152].

CSF liquid biopsy also has important failure modes. False-negative results may occur in tumors with limited CSF contact, low shedding, low tumor fraction, low-grade circumscribed growth, or post-treatment low-burden disease. Technical failure may arise from limited CSF volume, low nucleic-acid input, blood contamination, processing delays, storage variability, or differences among extraction, sequencing, and bioinformatic platforms. These constraints are particularly important in pediatric patients, where repeated sampling and adequate CSF volume may be difficult. Standardized thresholds for clinical interpretation also remain incomplete across tumor types and analyte classes. CSF results should therefore be interpreted as context-dependent adjunctive information: negative results do not exclude disease, and positive findings require integration with imaging, cytology, tissue data where available, treatment timing, and clinical status.

Heterogeneity between studies further complicates interpretation. Reported detection rates and clinical correlations vary according to tumor entity, disease stage, sampling route, timing relative to surgery or therapy, assay platform, sequencing depth, and the definition of molecular positivity. Some apparent discrepancies between studies may therefore reflect differences in pre-analytical workflow, tumor burden, or patient selection rather than true biological disagreement. Future studies should report these variables consistently and evaluate CSF biomarkers prospectively within harmonized, tumor-specific clinical contexts.

### 7.2. Diagnosis When Tissue Biopsy Is Unsafe or Anatomically Prohibitive

The clearest immediate clinical use is in tumors arising in eloquent or surgically hazardous sites, including the pons, cervicomedullary junction, optic pathway, hypothalamus, thalamus, and brainstem [152,153]. In such settings, the requirement for integrated molecular diagnosis under WHO CNS5 creates a practical problem when tissue is unsafe to obtain or unlikely to be adequate [44].

Here, CSF liquid biopsy can provide actionable molecular information without direct tumor sampling. In DMG, CSF ddPCR detection of H3K27M has shown high sensitivity and near-perfect specificity relative to tissue-confirmed status, supporting tissue-sparing diagnosis and trial eligibility in selected cases [90,140]. In inoperable *BRAF* V600E-mutant pLGG, CSF-based mutation detection has already been used to guide treatment with dabrafenib plus trametinib, including cases in which plasma was negative [135,136]. Broader targeted NGS platforms may further assist when single-target testing is insufficient, particularly in CSF-proximal tumors with adequate ctDNA burden [62,154]. At present, this application is best considered selective and still investigational.

### 7.3. Molecular Classification When Tissue Is Insufficient or Ambiguous

A second clinically important setting is the patient with tissue that is unavailable, exhausted, necrotic, poorly fixed, or otherwise inadequate for full molecular workup [44,153]. This problem has become more consequential as modern pediatric CNS tumor classification increasingly depends on methylation profiling, fusion status, and copy-number architecture.

In this context, CSF cfDNA may provide a complementary and investigational substrate for molecular profiling in selected patients when tissue is unavailable, exhausted, or insufficient for additional testing. In MB, CSF-based whole-genome bisulfite sequencing (WGBS), enzymatic methylation sequencing (EM-Seq), and cell-free reduced representation bisulfite sequencing (cfRRBS) have shown feasibility for subgroup assignment and distinction from related embryonal tumors using very low DNA input [7,119,120]. For fusion-driven entities such as ZFTA-fused ependymoma, CSF cfRNA has been proposed as a potential non-tissue route for confirming diagnostically essential fusions, although clinical validation remains incomplete [51]. Real-world precision-oncology experience further supports the feasibility of CSF cfDNA analysis at relapse when tumor tissue is unavailable or exhausted, including tumor detection by LP-WGS in selected cases [154]. However, the available evidence remains insufficient to support CSF cfDNA as a reliable replacement for tissue-based molecular classification. Current classifiers were developed primarily using tissue, and performance is likely to vary according to tumor type, tumor fraction, sampling compartment, pre-analytical workflow, and assay platform. Prospective validation is therefore required before CSF cfDNA can be used routinely as a substitute for tissue in this setting.

### 7.4. Baseline Risk Stratification

CSF liquid biopsy may also refine baseline risk assessment, although the strength of evidence differs by tumor type. In MB, postoperative CSF MRD positivity correlates with metastatic disease and adverse outcome, and molecular positivity may be present despite negative cytology, suggesting that CSF can identify biologically high-risk patients missed by conventional staging [63,118,155]. In DMG, baseline CSF H3K27M variant allele fraction correlates with MRI-defined tumor burden, although serial change appears more informative than a single baseline value [92,140].

In iGCTs, CSF already has an established baseline role through AFP and β-hCG, which directly inform diagnostic category and treatment intensity [24,147]. Emerging ctDNA data suggest that molecular profiling may complement this framework, particularly when conventional markers are equivocal [54]. In ependymoma, CSF detection of 1q gain may support baseline risk assignment when tissue copy-number profiling is unavailable, but this remains investigational [8]. At present, the most mature baseline applications are in MB and GCTs; elsewhere, prospective validation remains needed.

### 7.5. Measurable Residual Disease During and After Treatment

Among pediatric brain tumors, the strongest evidence for CSF liquid biopsy as an MRD tool comes from MB. In the SJMB03 analysis, post-treatment CSF MRD positivity predicted increased relapse risk, and molecular resurgence often preceded radiographic progression by months [63]. This identifies a potentially actionable window in which relapse-directed therapy might begin before overt radiographic failure. Early prospective support for this framework has also emerged in embryonal-tumor cohorts including MB and AT/RT [6].

In DMG and pHGG, the concept is related but not identical. Because these tumors are rarely reduced to a true minimal-residual state, serial CSF ctDNA functions less as binary MRD and more as a dynamic marker of tumor burden. In ONC201-treated DMG, sustained reduction in CSF H3K27M correlated with longer progression-free survival, whereas rising levels often preceded MRI-defined progression [92]. At present, however, MRD-guided management remains best confined to prospective protocols [63,156].

### 7.6. Relapse Detection and Augmentation of Radiographic Surveillance

MRI remains the backbone of surveillance in pediatric neuro-oncology, but it is limited in early leptomeningeal disease, post-treatment change, and equivocal progression [115,152]. CSF ctDNA can augment imaging by providing molecular evidence of recurrence, and in some cases, by detecting progression earlier than MRI.

In MB, resurgence of CSF ctDNA has preceded radiographic relapse in a meaningful subset of patients [63], and serial LP-WGS has documented genomic evolution at recurrence before or alongside clinical progression [121]. Similar early molecular recurrence has been observed in AT/RT [63]. In iGCTs, serial AFP and β-hCG are already standard surveillance tools, and ctDNA may add value in marker-negative or ambiguous cases [147,148]. A particularly important use case is the distinction between pseudo-progression and true progression. In DMG, stable or falling CSF H3K27M during equivocal MRI change has supported pseudo-progression, whereas rising ctDNA has favored true progression [92]. This remains one of the most promising near-term surveillance applications, although interpretation is still context dependent.

### 7.7. Treatment-Response Monitoring as a Pharmacodynamic Biomarker

CSF ctDNA also has emerging value as a pharmacodynamic marker, particularly when MRI is confounded by pseudo-response or pseudo-progression [92,114,151]. The strongest evidence again comes from ONC201-treated DMG, where CSF H3K27M kinetics correlated with progression-free survival and clarified ambiguous imaging findings; when combined with CSF metabolomic changes such as 2-hydroxyglutarate and glutamate, they provided a coherent signal of drug activity [92,146].

Parallel observations are emerging in other entities. In BRAF V600E-positive pLGG, conversion from detectable to undetectable CSF mutation signal has been observed during targeted therapy [136]. In MB, declining CNA signal accompanies effective treatment [63]. In NGGCT, ctDNA clearance during induction therapy may provide prognostic information beyond conventional protein-marker trends [146]. These applications remain promising but not yet fully standardized, and current guidance supports their use primarily in trials [115,157].

### 7.8. Clonal Evolution and Resistance Tracking

A final major application is non-invasive assessment of clonal evolution during treatment and at relapse. In pediatric brain tumors, repeat biopsy is often impractical, so clinical decisions are commonly based on archival molecular data that may no longer reflect the biology of recurrent disease. CSF ctDNA offers serial access to the evolving tumor genome and may reveal resistance mechanisms absent from the primary specimen [62,158].

In clinical sequencing studies, CSF ctDNA obtained at relapse has identified newly emergent alterations, including *PDGFRA*, *PIK3CA*, and other therapeutically relevant pathway changes not present in earlier tumor tissue [62,154,157]. Similar principles likely apply to MB, where acquired SMO mutations or CDK4/6-pathway alterations may have therapeutic implications at relapse [118,159]. Pediatric cohorts also suggest that CSF can capture genomic heterogeneity not fully represented by single-site tissue biopsy [42,62]. This application is best addressed with broad NGS strategies instead of single-target assays, ideally in a staged framework in which LP-WGS first confirms tumor-derived DNA, and deeper sequencing is applied once tumor fraction is sufficient [62,155,157]. Interpretation remains selective because CSF still provides a compartment-specific and incomplete view of total tumor heterogeneity.

Taken together, these seven decision points show that the clinical value of CSF liquid biopsy is context dependent. Its strongest current roles are tissue-sparing molecular diagnosis in selected tumors, MRD assessment in MB, pharmacodynamic monitoring in DMG, and molecular characterization at relapse when tissue is unavailable. The next phase of clinical translation will depend less on biological plausibility than on assay standardization, prospective trial embedding, and entity-specific definition of how CSF findings should alter management.

## 8. Barriers to Clinical Implementation and Future Directions

Despite strong biological rationale and rapidly expanding pediatric data, CSF liquid biopsy has not yet achieved routine clinical standardization. The principal barrier is no longer proof that tumor-derived signal can be recovered from CSF, but reproducibility across centers. In pediatric neuro-oncology, CSF is often a low-volume, low-input, and low-tumor-fraction specimen, such that collection site, timing relative to surgery or therapy, processing delay, fraction selection, storage, and blood contamination can materially alter biomarker yield and interpretation. These factors are now recognized as major determinants of assay performance rather than minor technical variables [5,6,160].

A second challenge is uneven analytical maturity across tumor types and analyte classes. CSF cfDNA/ctDNA currently has the strongest evidence base, particularly in MB, DMG, and selected GCTs, whereas cfRNA, EVs, proteomics, metabolomics, and CTC-based approaches remain less standardized and are supported mainly by smaller or discovery-stage studies. This heterogeneity reflects not only assay development, but also assay–biology mismatch: high-grade or CSF-adjacent tumors are generally more tractable, whereas low-shedding, low-grade, or anatomically secluded tumors remain more difficult to assess reliably [2,5,6,115,153].

Clinical interpretation frameworks are also underdeveloped. For most pediatric CNS tumors, there is still no consensus on actionable thresholds for positivity, molecular response, or MRD, and prospective validation has not kept pace with biomarker discovery. In addition, much of the literature remains retrospective and enriched for selected tumor types or specialized centers, limiting generalizability. These constraints are compounded by pediatric procedural and infrastructural realities: lumbar punctures are invasive, specimen volume is limited, repeat sampling may be difficult to justify, and implementation requires clinical-grade low-input workflows, molecular pathology support, bioinformatics capacity, and multicenter harmonization. At present, CSF liquid biopsy is therefore best understood as a complementary tool for diagnosis, risk refinement, pharmacodynamic monitoring, and relapse detection rather than as a stand-alone determinant of management in routine care [2,6,115].

**Figure 2:**
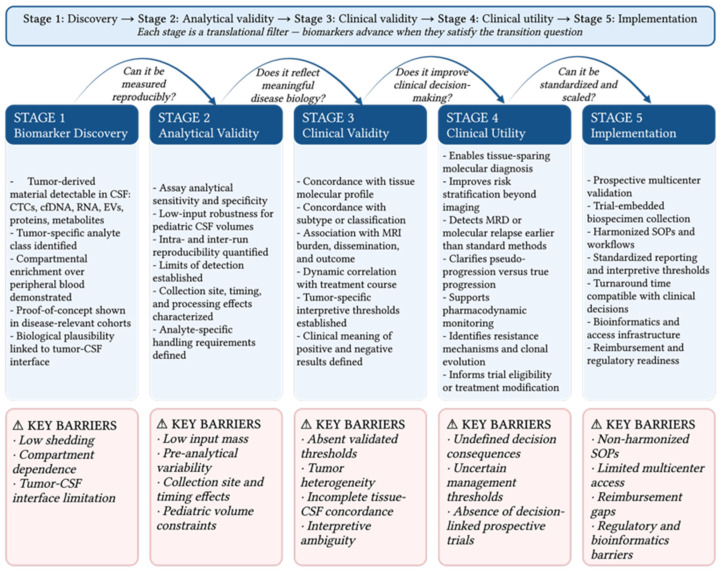
Translational roadmap for cerebrospinal fluid liquid biopsy in pediatric neuro-oncology: from biomarker discovery to clinical implementation. Created in BioRender. Asanova, A. (2026) https://BioRender.com/6239mci (accessed on 27 April 2026).

The most realistic future direction is not a single universal assay, but a tumor-matched and indication-specific model. The clearest near-term opportunities are MRD monitoring in MB, H3K27M tracking in DMG, tissue-sparing molecular diagnosis in selected BRAF-altered pediatric gliomas, and extension of established CSF biomarker frameworks in iGCTs. Future progress will likely depend on multi-analyte integration, clinically validated low-input workflows, and standardized reporting of pre-analytical variables so that CSF results can be interpreted within explicit clinical decision points and not as isolated molecular findings [3,5,6,152,153,161]. The main translational stages and bottlenecks that govern the movement of CSF biomarkers from discovery to clinical implementation are summarized in Figure 2.

### Implementation Requirements for Routine Clinical Translation

For CSF liquid biopsy to move from research use to routine pediatric neuro-oncology practice, clinical translation must be approached as a workflow problem rather than only as an assay-performance problem. The first requirement is standardization of specimen acquisition. Collection route, sampling timing, CSF volume, perioperative interval, treatment status, use of ventricular or shunt-derived specimens, and the presence of blood contamination should be documented, and where possible, harmonized across studies and clinical laboratories. This is particularly important in children, where samples are often low-volume, low-input, and difficult to repeat, and where small differences in handling may determine whether tumor signal is detectable at all [6,8,9,50,160].

The second requirement is analytical standardization. CSF liquid-biopsy assays will need defined minimum input requirements, validated extraction chemistries, acceptable limits for blood contamination and processing delay, standardized centrifugation and storage conditions, sequencing-depth requirements, and reproducible bioinformatic pipelines. For sequencing-based assays, clinically meaningful thresholds for variant allele fraction, CNA detection, methylation-classifier confidence, and longitudinal molecular change remain incompletely defined for most pediatric CNS tumor types. Without such thresholds, inter-platform comparison will remain difficult, and positive or negative findings may be interpreted inconsistently across centers [50,57,58,59,60,61,160].

The third requirement is clinical implementation evidence. Before routine adoption, CSF liquid-biopsy results should demonstrate incremental value over existing modalities, including tissue-based molecular pathology, MRI, conventional cytology, plasma-based assays, and standard clinical assessment. This requires prospective studies showing that CSF results can change diagnosis, risk stratification, treatment selection, response assessment, relapse detection, trial eligibility, or surveillance strategy. Finally, implementation will require reporting standards, clinically acceptable turnaround times, cost-effectiveness analyses, reimbursement pathways, and regulatory or clinical laboratory validation. Thus, the transition from research to practice will depend not only on whether CSF contains tumor-derived material, but on whether the entire workflow can produce reproducible, interpretable, affordable, and management-relevant results [151,160].

## 9. Conclusions

CSF liquid biopsy has moved beyond biological plausibility toward defined, dis-ease-specific clinical use cases in pediatric neuro-oncology. Its value is greatest where tumor anatomy, shedding biology, and analyte selection converge around a clear decision point. Rather than functioning as a universal assay platform, CSF provides disease- and decision-specific molecular information in settings where conventional tissue-based or imaging-based approaches remain limited.

The next phase of the field must focus on decision-linked validation rather than additional proof of principle. Progress will depend on harmonized pre-analytical work-flows, assay strategies matched to tumor biology, prospective multicenter studies with predefined clinical endpoints, and integration into cooperative-group trials. The key question is no longer simply whether CSF can detect tumor-derived material, but whether its use improves classification, refines risk, informs treatment monitoring, or alters management at clinically meaningful decision points. If these conditions are met, CSF liquid biopsy could become a practical component of precision care in pediatric neuro-oncology.

## Figures and Tables

**Table 1:** Conventional clinical applications of cerebrospinal fluid in pediatric central nervous system tumors.

Tumor Type	Current Standard CSF Use	Main Conventional Analyte	Role in Staging/Risk	Key Limitations
**Medulloblastoma** All molecular subgroups	LP for cytology, performed post-operatively (≥14 days) and at diagnosis; combined with neuraxis MRI for Chang staging	Cytology Protein/glucose	Positive cytology (M1) upstages to high-risk disease; drives craniospinal irradiation dose and adjuvant chemotherapy intensity	Cytology sensitivity ~30–70%; false negatives common. Pre-operative LP contraindicated (herniation risk). Post-surgical blood contamination confounds interpretation. Molecular subgroup not captured [11,12,25,26]
**Ependymoma** Intracranial & spinal	LP for leptomeningeal staging at diagnosis and relapse; routine use varies by center and spine MRI findings	Cytology	M-stage classification; positive CSF cytology shifts management toward craniospinal irradiation. Overall M+ rate low (~5–10%) at diagnosis	Very low clinical yield at diagnosis; cytology rarely positive when spine MRI is negative. Molecular subtype (e.g., *ZFTA*-fusion) not detectable by conventional CSF [13,14]
**CNS germ cell tumors** Germinoma & non-germinomatous	CSF tumor marker measurement is essential for diagnosis, disease classification, baseline risk assessment, and response assessment during treatment; LP also for cytology when clinically appropriate	AFP, β-hCG, PLAP (cytology)	Elevated CSF AFP or β-hCG supports diagnosis of non-germinomatous GCT and informs disease classification and treatment intensity; CSF-to-serum ratio may aid localization; marker decline or normalization during therapy supports response assessment	Normal markers do not exclude GCT, particularly pure germinoma. AFP elevation may be physiologic in infants < 1 year. CSF PLAP lacks standardization across laboratories. CSF tumor markers are not routinely used for surveillance after completion of therapy in all patients and should be interpreted according to protocol, tumor subtype, and clinical context [24,27,28].
**Diffuse midline glioma** H3 K27M-altered (incl. DIPG)	LP not routinely performed; diagnosis based on MRI ± biopsy. CSF cytology has no established staging role; LP may be contraindicated in brainstem location	None (standard)	No validated role in current standard of care; leptomeningeal dissemination occurs but is not routinely staged by CSF	LP carries herniation risk in brainstem/posterior fossa tumors. No biomarker approved for routine clinical CSF analysis. Emerging ctDNA/cfDNA approaches remain investigational [29,30]
**AT/RT** *SMARCB1*/*SMARCA4*-deficient	LP for cytology at diagnosis; leptomeningeal dissemination present in ~30% at diagnosis and drives upfront craniospinal therapy decisions	Cytology	M-stage is a key prognostic variable; M+ disease is associated with inferior survival and influences inclusion of craniospinal irradiation and intensified chemotherapy, including high-dose chemotherapy-based approaches	High false-negative rate of cytology despite radiologic dissemination. Very young age (most <3 years) complicates LP safety and CSF interpretation. Rapid disease progression limits utility of sequential sampling [15,16,31]
**Pediatric high-grade glioma** Non-DMG (e.g., HGG, GBM)	LP not routinely used for staging; performed if leptomeningeal spread suspected clinically or radiologically at relapse	Cytology (when performed)	No validated CSF-based staging system exists for pediatric non-DMG HGG/GBM. Leptomeningeal dissemination is uncommon at diagnosis but may occur during disease progression and is generally associated with poor prognosis	CSF cytology has limited sensitivity for sparse leptomeningeal tumor cells and may be negative despite radiologic leptomeningeal spread. LP may be unsafe in patients with mass effect, edema, or raised intracranial pressure. No tumor-specific CSF biomarker is currently used in routine care for pediatric non-DMG HGG/GBM [17,32]

**Abbreviations:** CSF, cerebrospinal fluid; AFP, alpha-fetoprotein; β-hCG, beta-human chorionic gonadotropin; LP, lumbar puncture; DMG, diffuse midline glioma; GBM, glioblastoma; GCT, germ cell tumors; HGG, high-grade glioma; PLAP, placental alkaline phosphatase.

**Table 2:** Biological factors influencing the likelihood of informative CSF tumor signal.

Factor	Expected Effect on CSF Signal	Example Tumor Contexts
**Ventricular/subarachnoid contact** Tumor interface with CSF compartment	↑ Signal ^1^ Direct shedding of tumor cells, DNA, proteins, and EVs into CSF is facilitated by physical proximity to the ependymal surface or subarachnoid space. Detection rates of ctDNA and tumor-derived protein are significantly higher when the tumor abuts a CSF-contiguous surface	Intraventricular ependymoma; choroid plexus tumors; tectal/pineal region tumors; third-ventricular craniopharyngioma [14,40,41]
**Leptomeningeal dissemination** M2–M3 spread	↑↑ Signal LMD provides a large, diffuse tumor-CSF interface, substantially amplifying shedding of all analyte classes (cells, ctDNA, protein, EVs). Detection sensitivity for liquid biopsy analytes peaks in the context of macroscopic dissemination. Even conventional cytology sensitivity rises with higher M-stage	Metastatic MB (M2–M3); AT/RT with spinal drop mets; disseminated CNS GCTs; leptomeningeal relapse of HGG [11,42,43]
**High-grade biology** Aggressive, invasive phenotype	↑ Signal Tumors with high mitotic activity, angiogenic disruption, and invasive growth patterns release more cfDNA and protein per unit mass. High-grade histology correlates with greater CSF ctDNA fraction independent of tumor location. Elevated CSF protein is more frequently observed in high- vs. low-grade pediatric CNS tumors	Pediatric GBM; H3 K27M-altered DMG; Group 3 MB; AT/RT; ETMR [30,37,44]
**Necrosis/high cellular turnover** Tumor cell death and lysis	↑ Signal Rapid tumor-cell turnover, treatment-related cell death, and necrotic tumor regions may increase release of fragmented DNA, intracellular proteins, and membrane-bound particles into the CSF compartment. This mechanism is most relevant in aggressive or treatment-exposed tumors, but the relationship between radiologic necrosis and measurable CSF signal remains context- and assay-dependent	GBM with central necrosis; progressive DMG/DIPG post-radiotherapy; post-treatment AT/RT; recurrent Group 3 MB [37,45]
**Low-grade circumscribed growth** Indolent, compact lesion	↓ Signal Slow proliferation and intact blood–brain/tumor barriers limit shedding into CSF. Cytology, ctDNA, and protein analytes are frequently undetectable or at background levels. The intact capsule of circumscribed tumors acts as a physical barrier to CSF egress. False-negative rates for all CSF analytes are highest in this category	Cerebellar pilocytic astrocytoma (WHO grade 1); DNET; ganglioglioma; hypothalamic pilocytic astrocytoma [46,47]
**Post-treatment low-burden disease** MRD/surveillance context	↓↓ Variable Following effective therapy, tumor mass and shedding both decrease, often rendering conventional analytes undetectable. Residual or recurrent micro-disease may escape cytology and imaging but may be detectable by ultrasensitive ctDNA or methylation-based approaches. Signal is highly dependent on residual tumor volume, clonal architecture, and the sensitivity of the assay platform	Post-HDCT MB surveillance; DIPG on ONC201; residual ependymoma after RT; AT/RT in maintenance phase [48,49]

^1^ The badges (↑↑, ↑, ↓, ↓↓/Variable) reflect the expected yield across all analyte classes. **Abbreviations:** AT/RT = atypical teratoid/rhabdoid tumor; CSF, cerebrospinal fluid; ctDNA, circulating tumor DNA; cfDNA, cell-free DNA; DIPG, diffuse intrinsic pontine glioma; DNET, dysembryoplastic neuroepithelial tumor; ETMR, embryonal tumor with multilayered rosettes; EVs, extracellular vesicles; HDCT, high-dose chemotherapy; LMD, leptomeningeal dissemination; MB, medulloblastoma.

**Table 3:** CSF analyte classes in pediatric brain tumors: information content, strengths, limitations, and current maturity.

Analyte Class	Principal Clinical/Research Utility	Major Strengths	Major Limitations	Pediatric CNS Tumors with Strongest Current Relevance	References
Conventional cytology	Detection of microscopic leptomeningeal dissemination; staging; risk assignment	Widely available; highly specific when clearly positive; already embedded in clinical pathways	Limited sensitivity; binary and morphologic; strongly influenced by timing, site, and specimen quality; no molecular information	MB, other embryonal tumors, ependymoma, AT/RT, iGCT	[17,18,20,22,26,64,65,66,67,68]
Routine CSF chemistry/established markers	Supportive assessment of dissemination; diagnosis and monitoring in iGCT	Rapid, inexpensive; AFP/β-hCG clinically actionable in iGCT	Mostly nonspecific outside iGCT; limited molecular resolution	iGCT	[24,69,70,71,72,73,74,75]
CTCs	Quantitative cell detection; potential single-cell genomics/transcriptomics; monitoring	Preserves intact cells; CSF CTC assays can exceed cytology sensitivity in epithelial leptomeningeal metastasis; potentially high information yield	Low yields in pediatric CNS tumors; EpCAM bias; highly handling-sensitive; no validated pediatric thresholds	Currently strongest conceptually in dissemination-prone tumors; pediatric primary CNS tumor application still limited	[3,38,62,76,77,78,79,80,81,82,83,84,85,86,87]
cfDNA/ctDNA—targeted assays (ddPCR, targeted NGS)	Mutation detection; actionable variant profiling; serial response monitoring	Highly sensitive for known variants; clinically actionable; feasible in low-input CSF	Target dependence; low shedding in some tumors; false negatives with low input or poor tumor-CSF contact	DMG (H3K27M), MB, selected pHGG and pLGG	[2,8,38,62,88,89,90,91,92,93,94,95]
cfDNA/ctDNA –LP-WGS/methylation workflows	MRD assessment; molecular classification; genome-wide profiling	Strong for CNA-rich tumors; can work at very low input; broad information yield	Requires optimized low-input workflows; lower sensitivity in low-shedding tumors; interpretation depends on tumor biology and compartment	Embryonal tumors, ependymoma, CNS GCT; broader pediatric cohorts	[6,8,9,10,57,63,96,97]
Cell-free RNA/miRNA	Expression-state profiling; response monitoring; classification support	Can reflect viable-cell states and pathway activity; biologically complementary to DNA	Pre-analytically fragile; lower standardization; mostly discovery-stage in pediatrics	MB, HGG, DIPG/DMG (emerging)	[5,98,99,100,101,102]
Extracellular vesicles (EVs)	Multi-layer biomarker discovery; mutation/amplification detection; proteomic and signaling readouts	Protects cargo; may enrich tumor-associated signal; multi-omics potential	Isolation and reporting heterogeneity; strong dependence on handling and workflow	Glioma framework established; pediatric MB and other tumors emerging	[59,103,104,105,106,107,108]
Proteins/proteomics	Candidate biomarker discovery; dissemination biology; personalized peptide tracking	Biologically interpretable; complements nucleic-acid assays	Cohort heterogeneity; age effects; limited reproducibility and validation	MB; selected pediatric CNS tumors in discovery cohorts	[108,109,110,111]
Metabolites/metabolomics	Diagnostic signatures; pharmacodynamic monitoring; multi-omics integration	Captures functional metabolic state; strong mechanistic relevance	Sensitive to pre-analytics and sampling context; limited pediatric validation	DMG pharmacodynamic studies; MB multi-omics; glioma framework	[50,99,112,113,114]

**Abbreviations:** AFP, alpha-fetoprotein; AT/RT, atypical teratoid/rhabdoid tumor; CNA, copy-number alteration; CSF, cerebrospinal fluid; CTC, circulating tumor cell; ctDNA, circulating tumor DNA; ddPCR, droplet digital PCR; DIPG, diffuse intrinsic pontine glioma; DMG, diffuse midline glioma; iGCT, intracranial germ cell tumor; LP-WGS, low-pass whole-genome sequencing; MB, medulloblastoma; MRD, measurable residual disease; NGS, next-generation sequencing; pHGG, pediatric high-grade glioma; pLGG, pediatric low-grade glioma; β-hCG, beta subunit of human chorionic gonadotropin.

**Table 4:** Integrated tumor-specific clinical-readiness framework for CSF liquid biopsy in pediatric brain tumors.

Tumor Type	Most Informative CSF Analyte(s)	Current Strongest Use Case	Best-Supported Molecular Target(s)	Sampling Feasibility/Procedural Burden	Current Level of Maturity/Clinical Readiness
Medulloblastoma [7,38,63,117,118,119,120,121,122,123,124,125]	cfDNA/ctDNA; methylation-based cfDNA profiling; targeted ddPCR	Molecular subgroup inference; MRD assessment; serial monitoring of relapse/evolution	*CTNNB1*; *PTCH1*/*BCOR*; monosomy 6; 9q loss; i17q; *MYC*/*MYCN* amplification	Often feasible because lumbar CSF is already obtained for staging, but postoperative timing, blood contamination, and repeated surveillance sampling remain important constraints	Most mature pediatric CSF ctDNA application. MRD and molecular monitoring are supported by strong translational evidence, but broad routine use still requires prospective standardization, entity-specific thresholds, and harmonized serial sampling protocols
AT/RT [63,117,126,127,128]	cfDNA/ctDNA; targeted sequencing; CNA profiling	Diagnostic clarification when cytology/imaging are equivocal; serial monitoring; relapse characterization	*SMARCB1* loss/mutation; chromosome 22q-associated loss; less commonly *SMARCA4*	Feasibility is limited by very young age, rapid disease course, need for sedation/anesthesia, and difficulty of repeated sampling. Opportunistic sampling during clinically indicated staging is most realistic	Biologically compelling but still early-stage. Current evidence is mainly case-based or derived from small embryonal-tumor cohorts; analytical thresholds, sampling intervals, and management-changing utility remain unstandardized
ETMR [129,130,131,132,133,134]	miRNA (especially CSF-enriched/CSF-relevant circulating miRNA); multi-omics CSF profiling; potentially cfDNA	Tumor-specific biomarker detection; future diagnosis/monitoring in ultra-rare disease	*C19MC* amplification; miR-517a; LIN28A-associated biology; minority *DICER1*-altered cases	Procedural feasibility is challenging because patients are often very young and disease is ultra-rare; CSF sampling is most defensible when clinically indicated or integrated into prospective protocols	Proof-of-concept/exploratory stage. The strongest signal comes from tumor-associated miRNA biology, but CSF-based analytical validity and clinical utility remain unvalidated
Pineoblastoma/rare embryonal tumors [9]	cfDNA/ctDNA; ultra-low-input sequencing	Molecular surveillance; differential diagnosis in pineal-region tumors; rare-tumor profiling	Tumor-associated CNA patterns; entity-specific rare molecular alterations depending on subtype	Feasibility depends on tumor location, staging needs, and access to diagnostic CSF; repeated sampling is limited by rarity, age, and uncertain management impact	Preliminary rare-disease stage. Evidence is mainly from mixed embryonal cohorts and real-world sequencing studies; tumor-specific validation, sampling feasibility, and clinical thresholds remain lacking
pLGG [8,9,42,94,135,136,137]	Targeted ctDNA/cfDNA; selected targeted NGS; potentially fusion-oriented cfRNA/RNA assays	Tissue-sparing molecular diagnosis in surgically inaccessible tumors; selected treatment selection	*BRAF* V600E; *KIAA1549*::*BRAF*; less often other MAPK-pathway alterations	Sampling is usually difficult to justify unless tissue biopsy is unsafe and molecular results would alter targeted therapy; low shedding increases false-negative risk	Selective clinical use/investigational. Potentially useful for detecting actionable drivers when tissue biopsy is unsafe or impractical, but limited by low shedding, low overall sensitivity, variable tumor–CSF contact, and risk of false-negative results
DMG/pHGG [8,9,62,90,92,114,135,136,137,138,139,140]	Targeted ctDNA/cfDNA (especially ddPCR); targeted NGS; complementary metabolomics	Serial molecular monitoring; pharmacodynamic response assessment; early progression detection; pseudo-progression adjudication	H3K27M; broader pHGG alterations including *BRAF* V600E, *IDH1*, *TP53*, *PIK3CA*, *PDGFRA* amplification	Procedural burden is substantial because LP may be unsafe or difficult in brainstem/posterior fossa disease; sampling is best when opportunistic, protocol-driven, or clearly linked to treatment monitoring	Strongest glioma evidence base. H3K27M CSF ctDNA monitoring is analytically promising and trial-integrated in selected contexts, but it is not yet a universal standard-of-care assay; safe sampling feasibility and validated longitudinal thresholds remain key barriers
Ependymoma [9,37,141,142,143,144]	cfDNA/ctDNA; CNA profiling; future cfRNA/EV-RNA for fusions	Detection of high-risk CNA states; serial monitoring at recurrence; future molecular classification support	1q gain (especially PFA); potential *ZFTA*::*RELA* and related *ZFTA* fusions by RNA-based assays	Feasible when CSF is already obtained for staging or relapse evaluation, but low diagnostic yield and variable shedding limit routine repeated sampling	Moderate biological rationale but limited clinical validation. The strongest near-term role is recurrence monitoring or high-risk CNA detection rather than routine diagnosis; prospective validation and fusion/CNA-specific workflows are still needed
iGCT [24,54,145,146,147,148]	Conventional CSF protein markers; cfDNA/ctDNA; metabolites; miRNAs	Diagnosis, risk stratification, treatment monitoring; extension of marker-based care with molecular layers	AFP, β-hCG; CNA patterns by ctDNA; emerging miR-371-373 cluster; metabolomic signatures	Often feasible in selected diagnostic contexts because CSF markers may already be clinically indicated; molecular extensions should be added only when they provide incremental value beyond AFP/β-hCG	Established conventional CSF biomarker framework for AFP/β-hCG. Molecular extensions using ctDNA, metabolites, and miRNAs are promising but remain emerging and are not yet routinely validated for standard clinical decision-making

**Note:** Sampling feasibility and clinical readiness should be interpreted in relation to tumor–CSF anatomy, expected shedding biology, procedural burden, need for sedation or anesthesia, feasibility of repeated sampling, availability of tissue or plasma alternatives, and whether the result is likely to change management. The maturity categories distinguish technical detectability from analytical validation and clinical utility. “Proof-of-concept” indicates that tumor-associated signal has been detected in CSF but remains exploratory. “Analytically promising” indicates reproducible detection in defined contexts, but not necessarily proven management benefit. “Clinical utility” requires evidence that the CSF result changes diagnosis, risk stratification, treatment selection, monitoring, relapse detection, or trial eligibility beyond existing methods. Sampling feasibility and clinical readiness should be interpreted in relation to tumor–CSF anatomy, expected shedding biology, procedural burden, availability of tissue or plasma alternatives, and whether the result is likely to change management. **Abbreviations:** AT/RT, atypical teratoid/rhabdoid tumor; cfDNA, cell-free DNA; CNA, copy-number alteration; CSF, cerebrospinal fluid; ctDNA, circulating tumor DNA; ddPCR, droplet digital PCR; DMG, diffuse midline glioma; ETMR, embryonal tumor with multilayered rosettes; iGCT, LP, lumbar puncture; intracranial germ cell tumor; MRD, measurable residual disease; NGS, next-generation sequencing; PFA, posterior fossa group A; pHGG, pediatric high-grade glioma; pLGG, pediatric low-grade glioma.

## Data Availability

No new data were created or analyzed in this study.

## Abbreviations

The following abbreviations are used in this manuscript:

AFP	Alpha-fetoprotein
AT/RT	Atypical teratoid/rhabdoid tumor
β-hCG	Beta subunit of human chorionic gonadotropin
BCOR	BCL6 corepressor
cfDNA	Cell-free DNA
cfRNA	Cell-free RNA
CNA	Copy-number alteration
CNS	Central nervous system
CSF	Cerebrospinal fluid
CTC(s)	Circulating tumor cell(s)
ctDNA	Circulating tumor DNA
ddPCR	Droplet digital PCR
DIPG	Diffuse intrinsic pontine glioma
DMG	Diffuse midline glioma
DNET	Dysembryoplastic neuroepithelial tumor
EM-seq	Enzymatic methylation sequencing
ETMR	Embryonal tumor with multilayered rosettes
EV(s)	Extracellular vesicle(s)
HGG	High-grade glioma
iGCT	Intracranial germ cell tumor
LDH	Lactate dehydrogenase
LMD	Leptomeningeal dissemination
LP	Lumbar puncture
LP-WGS	Low-pass whole-genome sequencing
MB	Medulloblastoma
miRNA	MicroRNA
MRI	Magnetic resonance imaging
MRD	Measurable residual disease
NGGCT	Non-germinomatous germ cell tumor
NGS	Next-generation sequencing
PFA	Posterior fossa group A
pHGG	Pediatric high-grade glioma
PLAP	Placental alkaline phosphatase
pLGG	Pediatric low-grade glioma
SHH	Sonic hedgehog
WHO	World Health Organization
WNT	Wingless-related integration site
